# Effect of *Qingjin Huatan* decoction on pulmonary function and inflammatory mediators in stable chronic obstructive pulmonary disease: A systematic review and meta-analysis

**DOI:** 10.1371/journal.pone.0322779

**Published:** 2025-05-07

**Authors:** Xuqin Du, Yan Li, Jiansen Wang, Youqin Jiang, Yaji Liu, Dingrong Zhang, Qi Wu, Shouhong Long

**Affiliations:** 1 School of Traditional Chinese Medicine, Chongqing University of Chinese Medicine, Chongqing, China; 2 Department of Emergency, Chongqing Bishan Hospital of Traditional Chinese Medicine, Chongqing, China; LAU Gilbert and Rose-Mary Chagoury School of Medicine: Lebanese American University School of Medicine, LEBANON

## Abstract

**Background:**

The inflammatory response is the main pathophysiological basis of stable chronic obstructive pulmonary disease (COPD). It is a key factor that leads to frequent exacerbations and disease progression. Suppressing the inflammatory response can improve pulmonary function, prognosis, and quality of life in stable COPD patients.

**Objective:**

To evaluate the effect of *Qingjin Huatan* decoction (QJHTD) on pulmonary function and inflammatory mediators in stable COPD patients.

**Methods:**

Randomized controlled trials (RCTs) on the treatment of stable COPD with QJHTD were retrieved from nine Chinese and English electronic databases up to June 30, 2024. The quality of the studies was assessed using the Cochrane Risk of Bias Tool and the modified Jadad scale. Statistical analysis, sensitivity analysis, and publication bias assessment were performed using Stata 17.0 software.

**Results:**

A total of 16 RCTs involving 1,228 stable COPD patients were included. Compared to standard treatment, QJHTD significantly improved pulmonary function, with increases in FEV1 (MD = 0.32, 95% CI [0.25, 0.38], *p *= 0.000), FVC (MD = 0.30, 95% CI [0.22, 0.37], *p *= 0.000), FEV1/FVC (MD = 5.58, 95% CI [4.81, 6.34], *p *= 0.000), and PaO_2_ (MD = 9.62, 95% CI [6.17, 13.08], *p *= 0.000), and a decrease in PaCO_2_ (MD = -9.12, 95% CI [–11.96, –6.28], *p *= 0.000). QJHTD also significantly suppressed the expression of inflammatory mediators, including TNF-α (MD = –7.47, 95% CI [–10.59, –4.34], *p *= 0.000), IL-6 (MD = -4.33, 95% CI [–6.17, –2.48], *p *= 0.000), and hs-CRP (MD = –9.11, 95% CI [–11.02, –7.20], *p *= 0.000). Additionally, QJHTD improved clinical efficacy (RR = 4.60, 95% CI [3.09, 6.86], *p *= 0.000) without increasing the incidence of adverse reactions (RR = 1.60, 95% CI [0.69, 2.46], *p* = 0.42).

**Conclusion:**

The current evidence suggests that QJHTD, as an adjunct therapy to standard treatment, may significantly improve pulmonary function, reduce inflammatory mediators, and enhance clinical efficacy in patients with stable COPD, with a favorable safety profile. However, these findings should be interpreted with caution due to several limitations, including small sample sizes, high heterogeneity among studies, and methodological weaknesses such as lack of blinding. More rigorously designed, high-quality, multicenter trials are needed to confirm these results.

## Introduction

Chronic obstructive pulmonary disease (COPD) is a widespread condition that poses a serious threat to human health. It significantly impacts the quality of life of patients and is a leading cause of mortality, imposing a substantial economic burden on patients, their families, and society [1]. COPD is a heterogeneous lung disease, characterized by progressively worsening airflow limitation and chronic respiratory symptoms such as cough, sputum production, and dyspnea [2]. The disease can be categorized into stable and acute exacerbation phases. Even during the stable phase, patients often experience cough, sputum production, and reduced pulmonary function [3]. These symptoms increase the risk of frequent exacerbations and hospitalizations. This results in significant economic consequences for individuals, healthcare systems, and society [4]. Smoking cessation, exercise, and pharmacotherapy can help prevent and control the frequency of acute COPD exacerbations and slow disease progression [5]. However, some patients with stable COPD still experience frequent acute exacerbations, severely affecting their quality of life and economic status [6]. Therefore, improving pulmonary function and reducing the frequency of acute exacerbations in stable COPD patients is a critical area of investigation. These interventions are vital for improving patients’ quality of life and reducing their economic burdens.

Persistent inflammatory responses are a crucial pathological mechanism in stable COPD patients [7]. Environmental factors such as smoke, dust, and pollutants can exacerbate airway inflammation. This process involves a range of inflammatory cells, cytokines, and mediators [8]. Current evidence confirms that ongoing inflammation is the primary mechanism behind acute exacerbations and disease progression in stable COPD [9]. It is closely associated with declines in pulmonary function and increased mortality [10]. Macrophages and neutrophils are the primary inflammatory cells involved in COPD development [11]. Upon exposure to cigarette smoke, macrophages get activated and release several inflammatory mediators, such as tumor necrosis factor-alpha (TNF-α) and interleukin-6 (IL-6) [11]. Concurrently, neutrophils accumulate in the lungs and become activated, worsening inflammation and airway remodeling [12]. Research by Bhatt et al. [13] demonstrated that TNF-α is involved in systemic inflammatory mechanisms and airway obstruction in COPD. Elevated levels of TNF-α are associated with impaired bronchial dilation in both COPD and asthma [14]. TNF-α knockout can reduce lung injury by activating the mitogen-activated protein kinase (MAPK) pathway, thereby slowing COPD progression [15]. IL-6 is a key cytokine in inflammation, and its persistent dysregulation plays a crucial role in acute systemic inflammatory responses and chronic immune-mediated diseases [16]. Serum IL-6 levels are closely related to pulmonary function; the more severe the pulmonary function impairment, the higher the serum IL-6 levels [17]. Thus, inhibiting inflammatory responses is a vital therapeutic strategy for reducing the frequency of acute exacerbations and improving the prognosis of stable COPD.

*Qingjin Huatan* Decoction (QJHTD, S1 Table) is a classic traditional Chinese medicine prescription. It originated in the Ming Dynasty and was formulated by physician Ye Wenling. The prescription is documented in the “*Genera Principles of Medicine*”[18]. It has been used for over 400 years to treat pulmonary diseases caused by phlegm-heat obstructing the lungs. Pharmacological research has confirmed that QJHTD has various therapeutic effects, including antitussive, expectorant, anti-inflammatory, antipyretic, and immune-enhancing properties [19]. Studies have shown that QJHTD downregulates the expression of p-STAT1, p-STAT3, p-JAK2 proteins, and JAK2 protein and gene expression, while upregulating SOCS3 protein and gene expression. This process inhibits inflammation and reduces lung tissue damage [20]. QJHTD also suppresses the expression of TLR4 and MUC5AC, decreases the levels of inflammatory factors in bronchoalveolar lavage fluid. Additionally, QJHTD improves mucus hypersecretion in the airways, and reduces the degree of airway inflammation [21]. QJHTD also inhibits the autophagy levels of airway epithelium [22]. In clinical practice, QJHTD is primarily used for stable COPD, community-acquired pneumonia, bronchitis, acute lung injury, and other pulmonary diseases [23]. Given the critical role of persistent inflammation in the acute exacerbations and disease progression of stable COPD. This study aims to explore the effects of QJHTD on pulmonary function and inflammatory mediators in stable COPD patients through a meta-analysis of randomized controlled trials (RCTs).

## Method

In our systematic review and meta-analysis, we followed the guidelines of the Preferred Reporting Items for Systematic Reviews and Meta-Analyses (PRISMA, S2 Table) [24]. We also registered our protocol with the International Prospective Register of Systematic Reviews (PROSPERO, CRD42024566457).

### Search strategy

We searched PubMed, Cochrane Library, Web of Science, Embase, Chinese Clinical Trial Registry, China National Knowledge Infrastructure (CNKI), China Science and Technology Journal Database (CQVIP), Wanfang Data, and China Biology Medicine Disc (CBM) for potential studies. The search period spanned from the inception of each database to June 30, 2024, with no language restrictions. Two reviewers (SL and YL) independently screened the included studies, and a third experienced reviewer (XD) combined the screening results through discussion with the two reviewers. The search utilized a combination of MeSH terms and keywords, including “chronic obstructive lung disease,” “chronic obstructive pulmonary disease,” “chronic obstructive airway disease,” “COPD,” “Qingjin Huatan Decoction,” “Qingjin Huatan Tang,” and “Qingjin Huatan Granules.” Additionally, we reviewed the reference lists of included studies to identify potential eligible trials. The detailed search strategy is provided in S1 Appendix.

### Eligibility criteria

The inclusion criteria were as follows: (1) Design: RCTs. (2) Participants: Participants with stable COPD diagnosed according to the Global Initiative for Chronic Obstructive Lung Disease (GOLD) guidelines or the COPD guidelines by the Chinese Medical Association Respiratory Disease Society. (3) Interventions: The control group received standard treatment (ST) recommended by guidelines, while the treatment group received ST plus QJHTD. (4) Outcome measures: Primary outcomes included pulmonary function (forced expiratory volume in one second [FEV1], forced vital capacity [FVC], and FEV1/FVC) or inflammatory mediators (TNF-α, IL-6, high-sensitivity C-reactive protein [hs-CRP]).

The exclusion criteria were as follows: (1) Non-RCTs. (2) Interventions containing other Chinese herbal preparations besides QJHTD. (3) Duplicate publications. (4) Basic research, reviews, conference abstracts, commentaries, and case reports. (5) Studies with insufficient data, where additional information could not be obtained after contacting the corresponding author via phone or email

### Data extraction

Two reviewers (JW and YJ) independently extracted data using standardized forms. Any discrepancies were resolved by consensus or discussion with a third reviewer (XD). Extracted information included the first author’s name, publication year, sample size, gender, age, disease duration, and interventions. Outcome measures analyzed were: (1) Pulmonary function: FEV1, FVC, FEV1/FVC, partial pressure of oxygen (PaO_2_), and partial pressure of carbon dioxide (PaCO_2_). (2) Inflammatory mediators: TNF-α, IL-6, hs-CRP. (3) Clinical efficacy and adverse reactions.

### Study quality assessment

Two reviewers (YL and DZ) assessed the quality of the included studies using the Cochrane risk of bias tool [25] and the modified Jadad scale [26]. The Cochrane risk of bias tool evaluates seven domains: random sequence generation, allocation concealment, blinding of participants and personnel, blinding of outcome assessment, incomplete outcome data, selective reporting, and other biases. The evaluation results are categorized as “low risk,” “high risk,” or “unclear.” The modified Jadad scale assesses four aspects: generation of random sequences, randomization concealment, blinding, and withdrawals/dropouts, scoring studies as 1–3 for low quality and 4–7 for high quality. Any disagreements were resolved by consensus or through discussion with a third reviewer (XD).

### Data analysis

Statistical analysis was performed with Stata 17.0 software. The odds ratio (OR) was used for categorical data, while the mean difference (MD) was used for continuous data as effect measures. Each effect measure was presented with its point estimate and 95% confidence interval (CI). Heterogeneity among study results was analyzed using the chi-squared test (with a significance level of α=0.1) and quantified with the *P*-value. If no statistical heterogeneity was found among the studies, a fixed-effect model was used for the meta-analysis. If statistical heterogeneity was present, the sources of heterogeneity were further analyzed. After excluding the influence of apparent clinical heterogeneity, a random-effects model was employed for the meta-analysis. Subgroup analysis or sensitivity analysis was used to address significant clinical heterogeneity. If necessary, a descriptive analysis was conducted. Publication bias was assessed using Egger’s and Begg’s tests.

## Results

### Study selection

**Fig 1:**
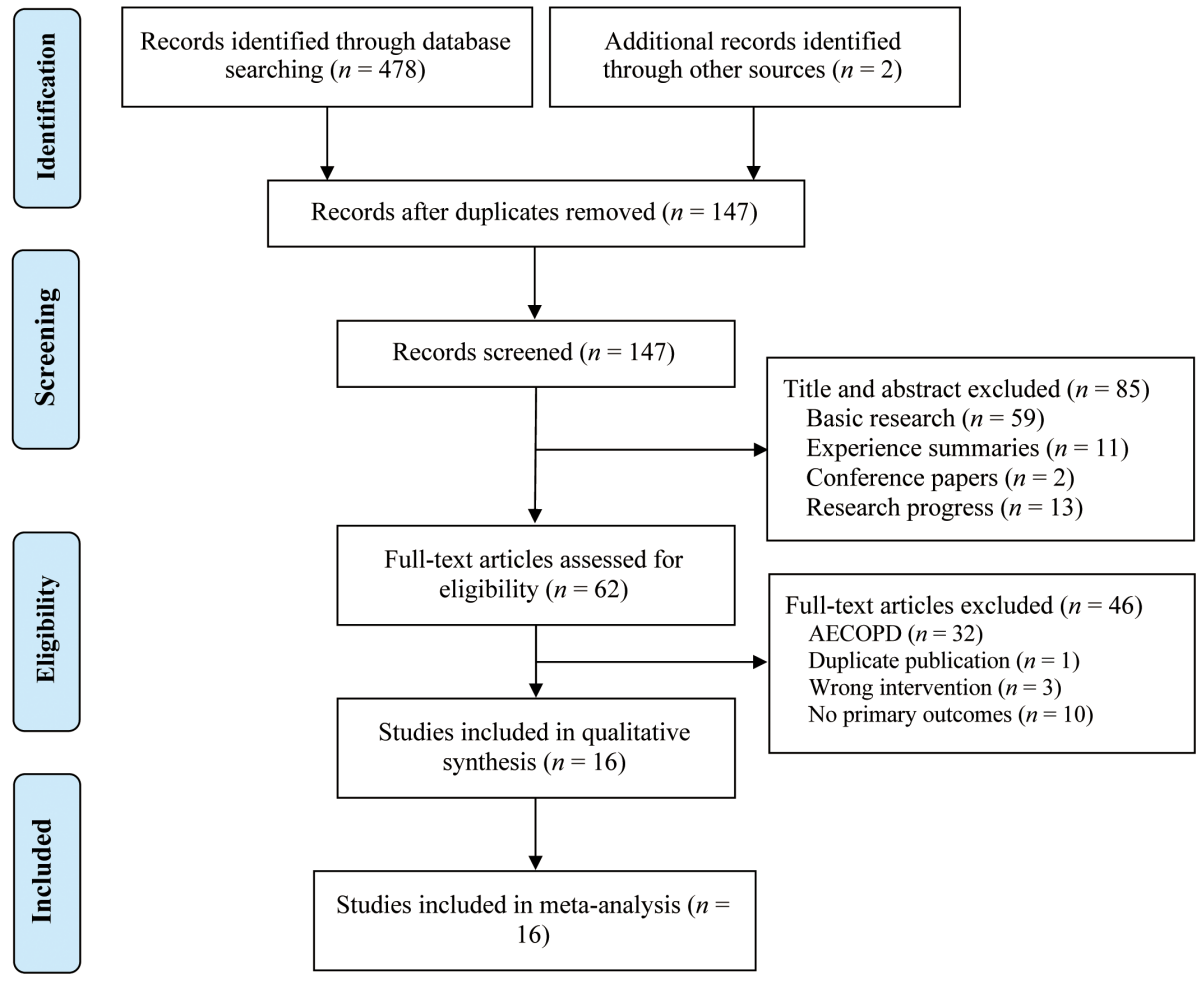
The PRISMA study flowchart.

The initial search yielded 480 relevant articles, distributed as follows: PubMed (*n *= 6), Embase (*n *= 3), Cochrane Library (*n *= 4), Web of Science (*n *= 3), CNKI (*n *= 128), CBM (*n *= 109), CQVIP (*n *= 93), WanFang Data (*n *= 133), and Chinese Clinical Trial Registry (*n *= 2). After screening titles and abstracts, 53 articles were excluded. Following a full-text review, 46 articles were further screened, resulting in the final inclusion of 16 RCTs [27–42] comprising a total of 1,228 participants. The selection process of the studies is illustrated in Fig 1.

### Characteristics of included studies

The basic characteristics of the included studies are shown in Table 1. A total of 16 RCTs were included. All conducted in China, involving 1,228 stable COPD patients (724 males and 504 females). These studies were published between 2011 and 2023, with sample sizes ranging from 25 to 60. The control groups received standard treatment recommended by GOLD or the Chinese Medical Association Respiratory Disease Society COPD guidelines, including oxygen therapy, anti-infective measures, cough relief, and bronchodilation. The treatment groups received a combination of QJHTD plus standard treatment. The outcomes reported in the included studies were as follows: FEV1 [30,32–35,40,41], FVC [30,32–35,40,41], FEV1/FVC [27,28,30–36,38–41], PaO_2_ [28,29,38,40,42], and PaCO_2_ [28,29,38,40,42]. Additionally, the studies reported on TNF-α [29,35,36,38], IL-6 [29,31,35–38], hs-CRP [31,35–38], clinical efficacy [27,29–32,34–42], and adverse reactions [27,29–31,34,35].

**Table 1.** Included studies basic characteristics.

Study ID	Sample size		Sex (M/F)		Mean age (years)		COPD course (years)		Interventions		ST drugs	Treatment duration	Outcomes
T	C	T	C	T	C	T	C	T	C			
Cai et al. (2015)	32	32	17/15	20/12	55.1	51.3	16.8	14.9	QJHTD + ST	ST	ICS + LABA	28d	③⑨⑩
Hua. (2020)	45	45	26/19	25/20	70.5 ± 5.1	70.1 ± 4.9	–	–	QJHTD + ST	ST	ICS + LABA + Ambroxol	28d	③④⑤
Huang et al. (2020)	25	25	12/13	13/12	54.01 ± 5.56	53.78 ± 5.67	7.74 ± 1.95	7.57 ± 1.58	QJHTD + ST	ST	ICS + LABA	14d	④⑤⑥⑦⑨⑩
Li and Wang. (2021)	42	42	27/15	28/14	73.5 ± 1.6	73.6 ± 1.8	5.35 ± 1.28	5.58 ± 1.46	QJHTD + ST	ST	ICS + LABA + Sustained-release theophylline tablets + Carbocisteine tablets	10d	①②③⑨⑩
Lin and Chen. (2016)	33	33	19/14	18/15	65.3 ± 6.09	66.0 ± 5.34	–	–	QJHTD + ST	ST	ICS + LABA + Ambroxol	28d	③⑦⑧⑨⑩
Qi. (2021)	46	46	24/22	23/23	61.1 ± 5.4	60.3 ± 6.1	2.9 ± 0.6	3.0 ± 0.7	QJHTD + ST	ST	ICS + LABA + Sustained-release theophylline tablets	28d	①②③⑨
Sun and Xu. (2020)	50	50	29/21	28/22	53.15 ± 12.61	52.61 ± 13.29	6.73 ± 2.35	6.52 ± 2.18	QJHTD + ST	ST	ICS + LABA + Sustained-release theophylline tablets	10d	①②③
Wan. (2019)	30	30	18/12	20/10	56.8 ± 12.8	53.9 ± 11.2	8	7	QJHTD + ST	ST	ICS + LABA + Sustained-release theophylline tablets	28d	①②③⑨⑩
Wang. (2023)	39	39	24/15	26/13	75.14 ± 2.04	75.23 ± 2.10	–	–	QJHTD + ST	ST	ICS + LABA + Ambroxol	14d	①②③⑥⑦⑧⑨⑩
Wu et al. (2023)	30	30	18/12	17/13	64.70 ± 5.50	7.86 ± 2.54	64.50 ± 6.32	8.23 ± 2.46	QJHTD + ST	ST	ICS + LABA	28d	③⑥⑦⑧⑨
Xia. (2021)	44	44	41/3	40/4	70.25 ± 3.69	70.18 ± 3.47	9.25 ± 0.63	9.18 ± 0.71	QJHTD + ST	ST	ICS + LABA + SAMA + Ambroxol	28d	⑦⑧⑨
Yi et al. (2018)	60	60	30/30	29/31	70.83 ± 7.96	73.95 ± 7.52	–	–	QJHTD + ST	ST	ICS + LABA + Ambroxol	21d	③④⑤⑥⑦⑧⑨
Yu et al. (2015)	27	25	14/13	13/12	62.45 ± 5.32	65.00 ± 1.98	9.12 ± 4.17	9.66 ± 6.11	QJHTD + ST	ST	ICS + LABA + Sustained-release theophylline tablets + Ambroxol	14d	③⑨
Zhang and Zhang. (2015)	35	30	20/15	16/14	62.37 ± 12.68	61.22 ± 12.63	12.85 ± 3.79	14.33 ± 4.20	QJHTD + ST	ST	ICS + LABA	14d	①②③④⑤⑨
Zhang. (2011)	34	33	20/14	18/15	60.8	58.9	10.7	9.8	QJHTD + ST	ST	ICS + LABA	14d	③⑨
Zou et al. (2020)	46	46	26/20	25/21	59.93 ± 2.49	59.89 ± 2.46	5.37 ± 1.09	5.34 ± 1.08	QJHTD + ST	ST	ICS + LABA + LAMA	14d	④⑤⑨

Note: C, control group; T, treatment group; M, male; F, female; COPD, chronic obstructive pulmonary disease; QJHTD, *Qingjin Huatan* decoction; ST, standard treatment recommended by GOLD or the Chinese Medical Association Respiratory Disease Society COPD guidelines; ICS: inhaled corticosteroids; LABA: long-acting β_2_-agonists; LAMA: long-acting muscarinic antagonists; Outcomes: ①FEV1; ②FVC; ③FEV1/FVC; ④PaO_2_; ⑤PaCO_2_; ⑥TNF-α; ⑦IL-6; ⑧hs-CRP; ⑨Clinical efficacy; ⑩Adverse reactions. -, not reported.

### Quality assessment of included studies

**Fig 2:**
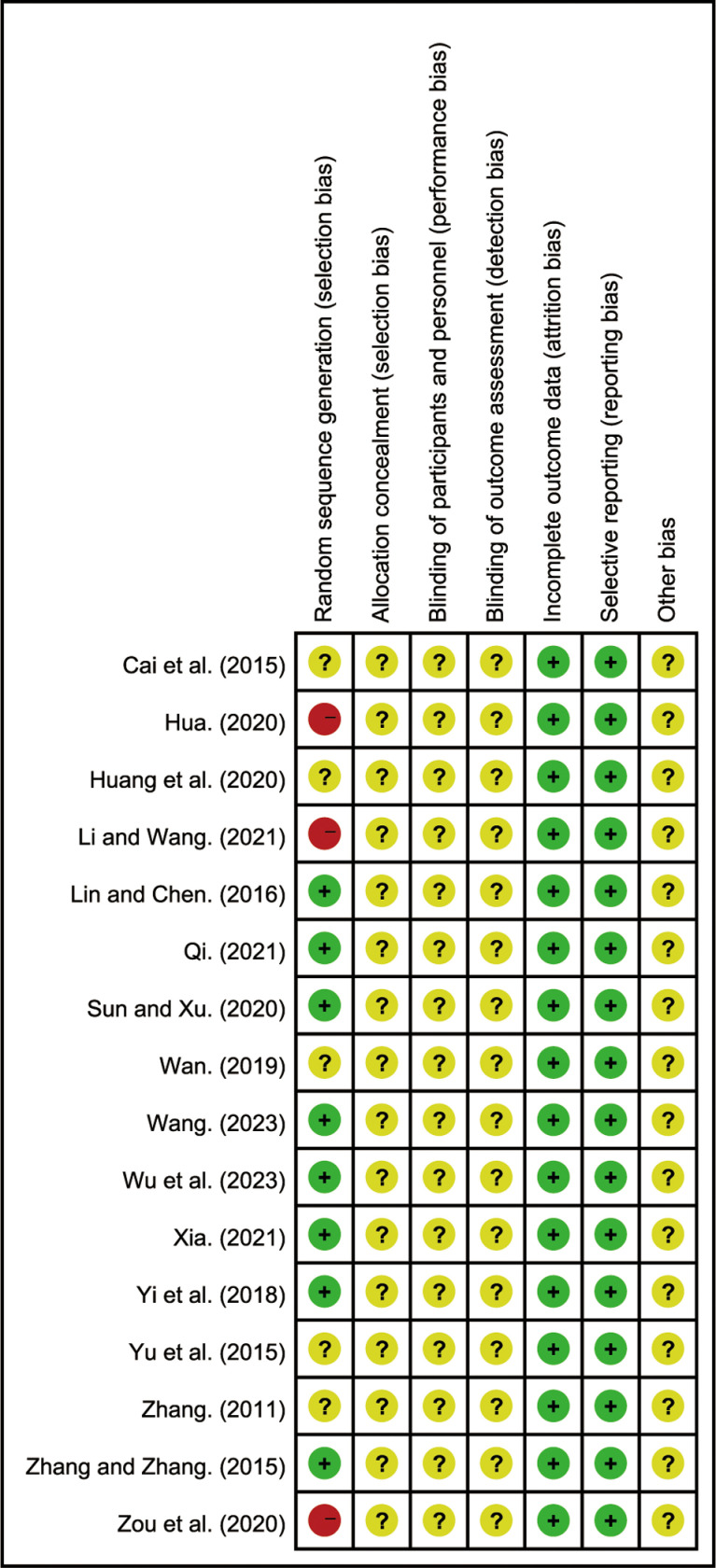
Bias risk assessment of included studies.

Among the 16 RCTs, 8 studies [31–33,35–38,40] used random number tables for allocation and were assessed as low risk of bias. 3 studies [28,30,42] grouped participants based on medication differences and were assessed as high risk. None of the studies reported blinding or allocation concealment, thus they were rated as unclear risk. All studies did not selectively report outcomes and were rated as low risk for selective reporting. The sources of other biases in all studies were unclear. The risk of bias assessment is shown in Fig 2 and S3 Table.

### Primary outcomes

#### FEV1.

**Fig 3:**
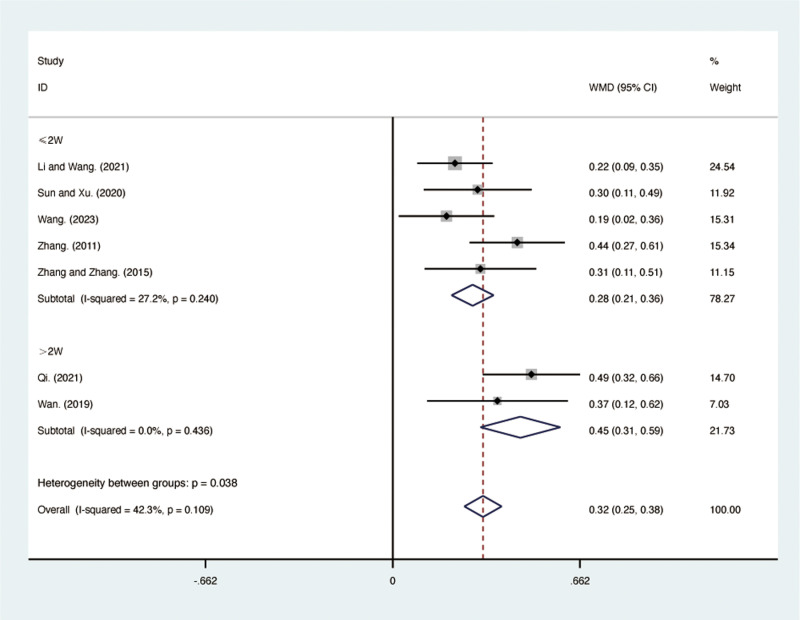
Forest plot for FEV1.

Seven RCTs [30,32–35,40,41], including 546 patients, reported FEV1 with low heterogeneity among the included studies (*I*² = 42.3%, *p* = 0.109). A fixed-effects model was used for the meta-analysis. The results showed that compared to ST, QJHTD significantly improved FEV1 in patients with stable COPD (MD = 0.32, 95% CI [0.25, 0.38], *p *= 0.000, Fig 3). Subgroup analysis based on the treatment duration indicated significant differences between QJHTD and ST: less than 2 weeks (MD = 0.28, 95% CI [0.21, 0.36], *p* = 0.000, Fig 3) and more than 2 weeks (MD = 0.45, 95% CI [0.31, 0.59], *p* = 0.000, Fig 3).

#### FVC.

**Fig 4:**
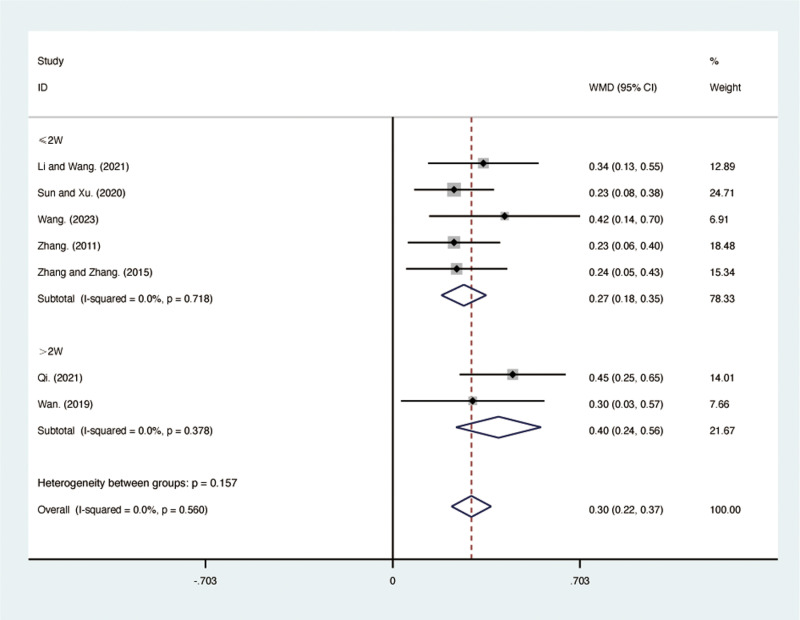
Forest plot for FVC.

Seven RCTs [30,32–35,40,41], including 546 patients, reported FVC with low heterogeneity among the included studies (*I*² = 0.0%, *p* = 0.560). A fixed-effects model was used for the meta-analysis. The results showed that compared to ST, QJHTD significantly improved FVC in patients with stable COPD (MD = 0.30, 95% CI [0.22, 0.37], *p *= 0.000, Fig 4). Subgroup analysis based on the treatment duration indicated significant differences between QJHTD and ST: less than 2 weeks (MD = 0.27, 95% CI [0.18, 0.35], *p* = 0.000, Fig 4) and more than 2 weeks (MD = 0.40, 95% CI [0.24, 0.56], *p* = 0.000, Fig 4).

#### FEV1/FVC.

**Fig 5:**
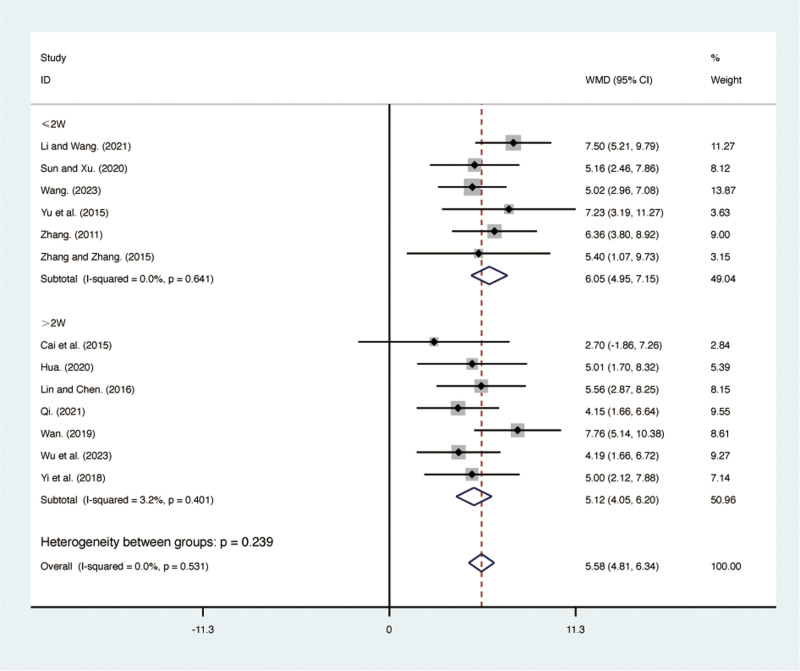
Forest plot for FEV1/FVC.

Thirteen RCTs [27,28,30–36,38–41], including 998 patients, reported FEV1/FVC with low heterogeneity among the included studies (*I*² = 0.0%, *p* = 0.531). A fixed-effects model was used for the meta-analysis. The results showed that compared to ST, QJHTD significantly improved FEV1/FVC in patients with stable COPD (MD = 5.58, 95% CI [4.81, 6.34], *p *= 0.000, Fig 5). Subgroup analysis based on the treatment duration indicated significant differences between QJHTD and ST: less than 2 weeks (MD = 6.05, 95% CI [4.95, 7.15], *p* = 0.000, Fig 5) and more than 2 weeks (MD = 5.12, 95% CI [4.05, 6.20], *p* = 0.000, Fig 5).

#### PaO_2_.

**Fig 6:**
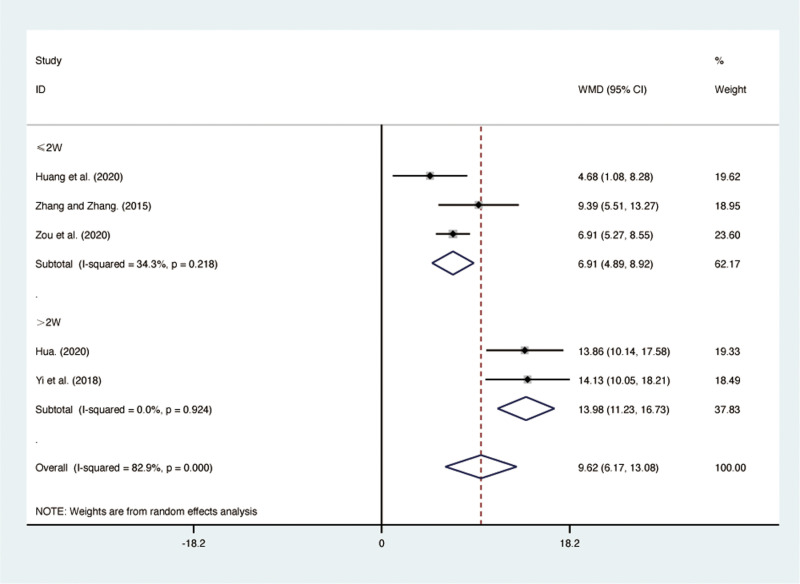
Forest plot for PaO_2_.

Five RCTs [28,29,38,40,42], including 417 patients, reported PaO_2_ with high heterogeneity among the included studies (*I*² = 82.9%, *p* = 0.000). A random-effects model was used for the meta-analysis. The results showed that compared to ST, QJHTD significantly improved PaO_2_ in patients with stable COPD (MD = 9.62, 95% CI [6.17, 13.08], *p *= 0.000, Fig 6). Subgroup analysis based on the treatment duration indicated significant differences between QJHTD and ST: less than 2 weeks (MD = 6.91, 95% CI [4.89, 8.92], *p* = 0.000, Fig 6) and more than 2 weeks (MD = 13.98, 95% CI [11.23, 16.73], *p* = 0.000, Fig 6).

#### PaCO_2_.

**Fig 7:**
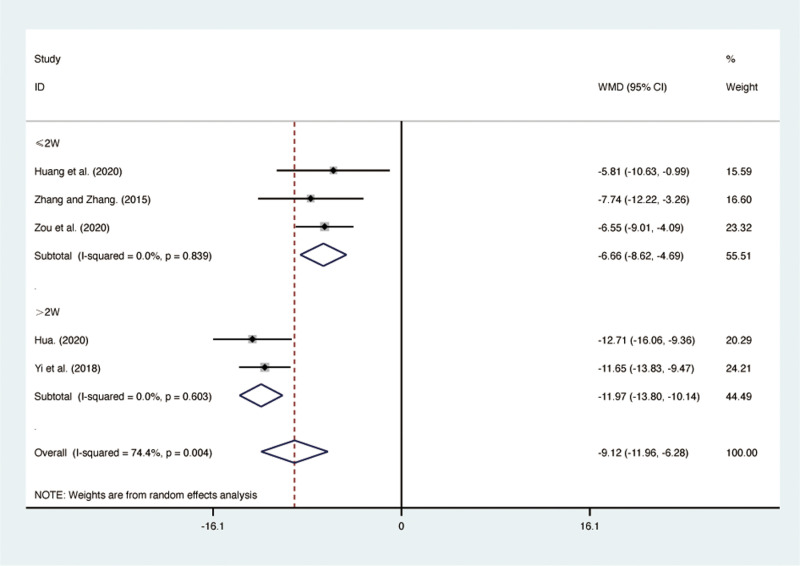
Forest plot for PaCO_2_.

Five RCTs [28,29,38,40,42], including 417 patients, reported PaCO_2_ with high heterogeneity among the included studies (*I*² = 74.4%, *p* = 0.004). A random-effects model was used for the meta-analysis. The results showed that compared to ST, QJHTD significantly reduced PaCO_2_ in patients with stable COPD (MD = -9.12, 95% CI [-11.96, -6.28], *p *= 0.000, Fig 7). Subgroup analysis based on the treatment duration indicated significant differences between QJHTD and ST: less than 2 weeks (MD = -6.66, 95% CI [-8.62, -4.69], *p* = 0.000, Fig 7) and more than 2 weeks (MD = -11.97, 95% CI [-13.80, -10.14], *p* = 0.000, Fig 7).

#### TNF-*α.*

**Fig 8:**
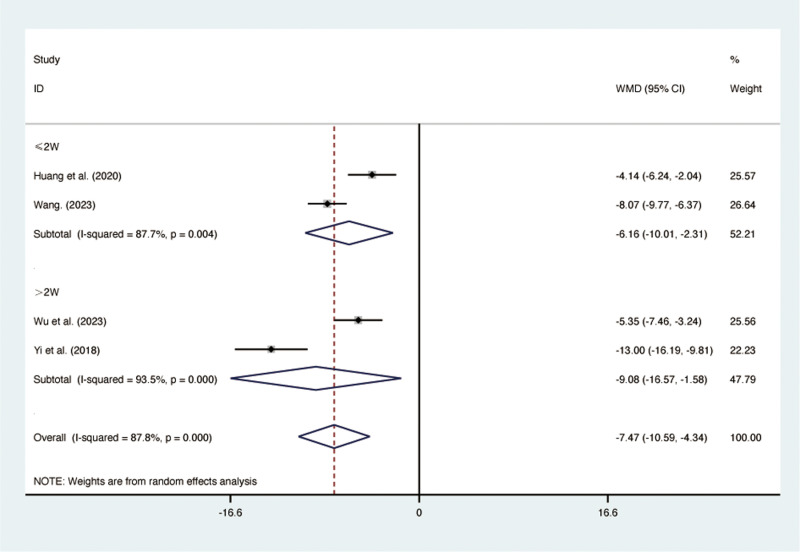
Forest plot for TNF- α.

Four RCTs [29,35,36,38], including 308 patients, reported TNF-α with high heterogeneity among the included studies (*I*² = 87.7%, *p* = 0.000). A random-effects model was used for the meta-analysis. The results showed that compared to ST, QJHTD significantly reduced TNF-α in patients with stable COPD (MD = -7.47, 95% CI [-10.59, -4.34], *p *= 0.000, Fig 8). Subgroup analysis based on the treatment duration indicated significant differences between QJHTD and ST: less than 2 weeks (MD = -6.16, 95% CI [-10.01, -2.31], *p* = 0.000, Fig 8) and more than 2 weeks (MD = -9.08, 95% CI [-16.57, -1.58], *p* = 0.000, Fig 8).

#### IL-6.

**Fig 9:**
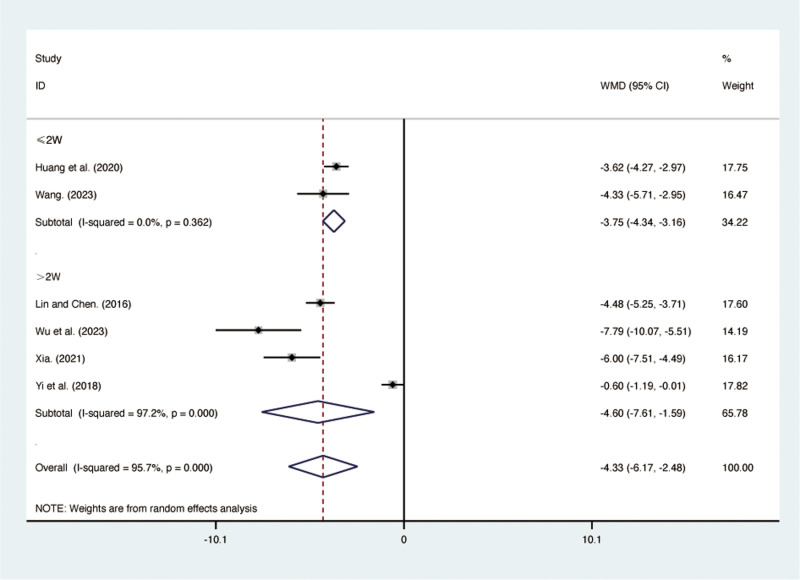
Forest plot for IL-6.

Six RCTs [29,31,35–38], including 462 patients, reported IL-6 with high heterogeneity among the included studies (*I*² = 95.7%, *p* = 0.000). A random-effects model was used for the meta-analysis. The results showed that compared to ST, QJHTD significantly reduced IL-6 in patients with stable COPD (MD = -4.33, 95% CI [-6.17, -2.48], *p *= 0.000, Fig 9). Subgroup analysis based on the treatment duration indicated significant differences between QJHTD and ST: less than 2 weeks (MD = -3.75, 95% CI [-4.34, -2.16], *p* = 0.000, Fig 9) and more than 2 weeks (MD = -4.60, 95% CI [-7.61, -1.59], *p* = 0.000, Fig 9).

#### hs-CRP.

**Fig 10:**
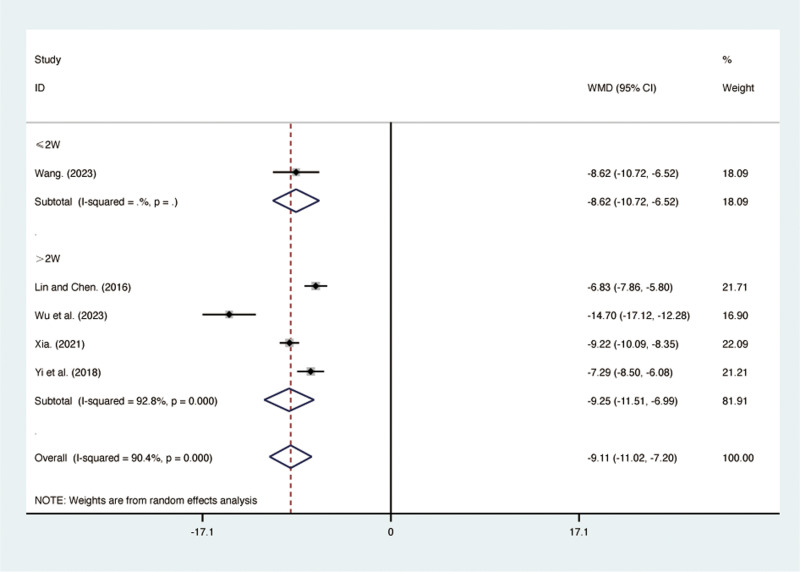
Forest plot for hs-CRP.

Five RCTs [31,35–38], including 412 patients, reported hs-CRP with high heterogeneity among the included studies (*I*² = 90.4%, *p* = 0.000). A random-effects model was used for the meta-analysis. The results showed that compared to ST, QJHTD significantly reduced hs-CRP in patients with stable COPD (MD = -9.11, 95% CI [-11.02, -7.20], *p *= 0.000, Fig 10). Subgroup analysis based on the treatment duration indicated significant differences between QJHTD and ST: less than 2 weeks (MD = -8.62, 95% CI [-10.72, -6.52], *p* = 0.000, Fig 10) and more than 2 weeks (MD = -9.25, 95% CI [-11.51, -6.99], *p* = 0.000, Fig 10).

### Secondary outcomes

#### Clinical efficacy.

**Fig 11:**
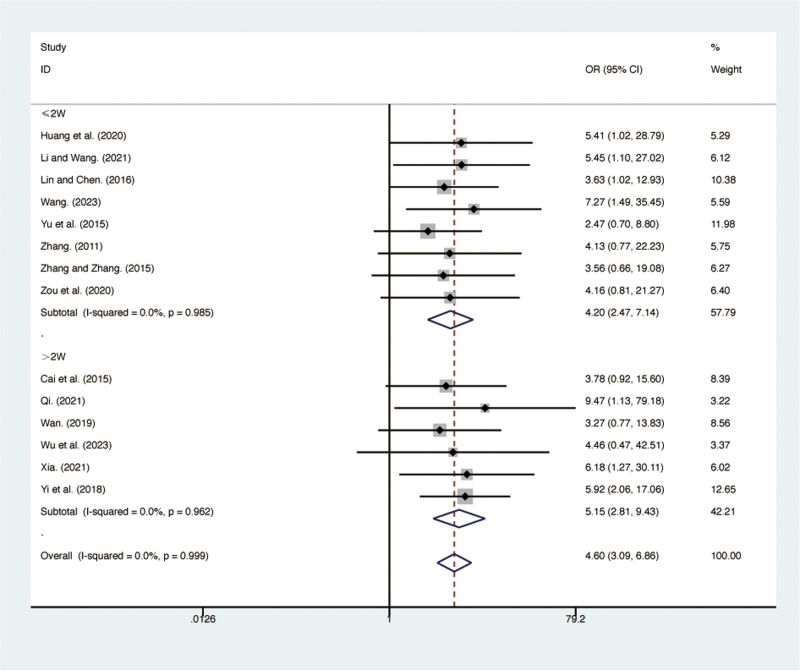
Forest plot for clinical efficacy.

Fourteen RCTs [27,29–32,34–42], including 1,036 patients, reported clinical efficacy with low heterogeneity among the included studies (*I*² = 0.0%, *p* = 0.999). A fixed-effects model was used for the meta-analysis. The results showed that compared to ST, QJHTD significantly improved clinical efficacy in patients with stable COPD (RR = 4.60, 95% CI [3.09, 6.86], *p *= 0.000, Fig 11). Subgroup analysis based on the treatment duration indicated significant differences between QJHTD and ST: less than 2 weeks (RR = 4.20, 95% CI [2.47, 7.14], *p* = 0.000, Fig 11) and more than 2 weeks (RR = 5.15, 95% CI [2.81, 9.43], *p* = 0.000, Fig 11).

#### Adverse reactions.

Among the 16 included studies, only 6 studies [27,29–31,34,35] reported adverse reactions, including drowsiness, insomnia, nausea, vomiting, dizziness, palpitations, rash, and elevated blood glucose. However, the pooled results indicated no significant difference in adverse reactions between the two groups (RR = 1.60, 95% CI [0.69, 2.46], *p* = 0.42). Detailed adverse reactions are provided in Table 2.

**Table 2.** The incidence rate of adverse reactions.

Adverse reaction symptoms	Study ID	The number of adverse reactions	
T	C
Drowsiness	Li and Wang. (2021)	2	1
Insomnia	Wan. (2019)	2	3
Nausea	Li and Wang. (2021); Li and Wang. (2021); Lin and Chen. (2016); Wan. (2019); Wang. (2023)	8	5
Vomiting	Lin and Chen. (2016); Wan. (2019)	1	2
Dizziness	Huang et al. (2020); Li and Wang. (2021); Lin and Chen. (2016)	4	3
Palpitations	Wan. (2019); Wang. (2023)	2	1
Rash	Huang et al. (2020); Wang. (2023)	1	2
Elevated blood glucose	Cai et al. (2015)	4	2
Total reactions	－	24/201	19/201
Incidence rate	－	11.94%	9.45%

### Sensitivity analysis

**Fig 12:**
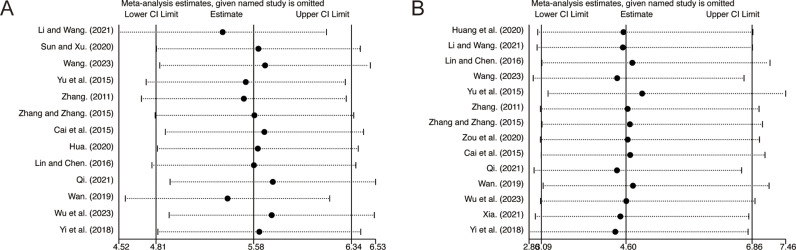
The results of sensitivity analysis: (A) FEV1/FVC. (B) Clinical efficacy.

Sensitivity analysis was performed to assess the reliability and robustness of the results by excluding individual studies one at a time, including FEV1/FVC (Fig 12A) and clinical efficacy (Fig 12B). The findings demonstrated that the removal of any single study did not significantly alter the pooled outcomes, indicating that the combined results are robust and reliable.

### Publication bias

**Fig 13:**
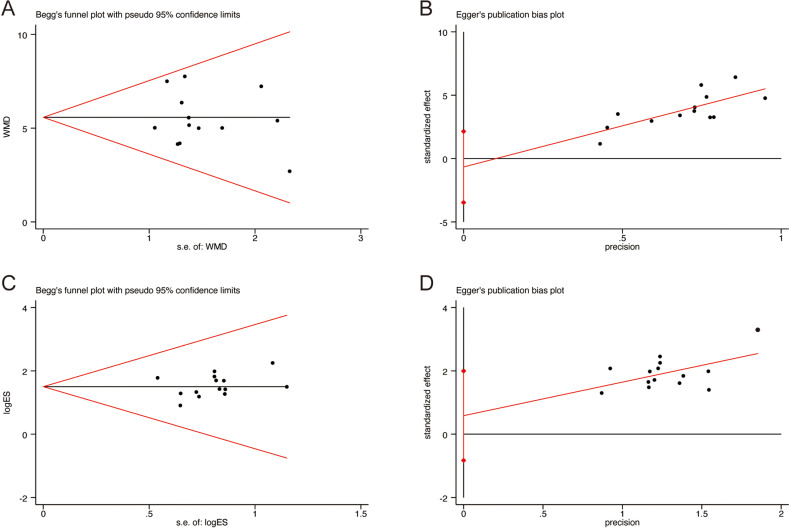
The results of publication bias: (A) Begg’s funnel plot of FEV1/FVC; (B) Egger’s funnel plot of FEV1/FVC; (C) Begg’s funnel plot of clinical efficacy; (D) Egger’s funnel plot of clinical efficacy.

Publication bias was evaluated using Begg’s and Egger’s tests. The results indicated no significant publication bias for FEV1/FVC (Begg’s test: *p* = 0.760, Fig 13A; Egger’s test: *p* = 0.612, Fig 13B) and clinical efficacy (Begg’s test: *p* = 0.381, Fig 13C; Egger’s test: *p* = 0.385, Fig 13D).

## Discussion

COPD is a significant global public health concern and is currently the third leading cause of death worldwide [1]. The etiology and pathogenesis of COPD are highly complex. These involve a combination of psychological and genetic factors, along with their interactions with environmental influences such as air pollution and smoking [43].

The characteristic pathological changes in COPD include chronic inflammation of the airways, lung parenchyma, and pulmonary vasculature. Other changes include airway wall remodeling and destruction of the lung parenchyma [44]. Together, these changes form the pathological basis for persistent airflow limitation. Airway inflammation is central to the pathogenesis of COPD and plays a crucial role throughout the disease’s progression [45]. Suppressing inflammatory responses is essential for improving pulmonary function, reducing the frequency of acute exacerbations, and enhancing the quality of life for COPD patients [46,47].

QJHTD is a classic traditional Chinese medicine formula. It has been used in China for over 400 years to treat pulmonary diseases, particularly COPD. Animal studies have demonstrated that QJHTD significantly inhibits various inflammatory factors, such as TNF-α, IL-6, and hs-CRP [48,49]. Given the critical role of inflammation in the pathogenesis and progression of COPD, this study aims to perform a meta-analysis of RCTs. The goal is to systematically evaluate the impact of QJHTD on pulmonary function and inflammatory mediators in patients with stable COPD. The results of this analysis are intended to provide more reliable evidence for clinical decision-making in the treatment of stable COPD.

### Summary of main results

This meta-analysis, based on RCTs, demonstrates the efficacy of QJHTD in treating patients with stable COPD. The analysis focuses on its effects on pulmonary function and inflammatory mediators. Sixteen RCTs with a total of 1,228 patients were included in the analysis. Compared to standard treatment, QJHTD significantly improved pulmonary function in stable COPD patients. This improvement was evident from increases in FEV1, FVC, FEV1/FVC, and PaO2, and a reduction in PaCO2. Additionally, QJHTD reduced inflammatory responses in stable COPD patients. It lowered the levels of inflammatory mediators such as TNF-α, IL-6, and hs-CRP. Furthermore, QJHTD enhanced clinical efficacy and demonstrated good safety in patients with stable COPD.

### Comparison with previous studies

Current pharmacological treatments for COPD primarily rely on bronchodilators (LABA/LAMA), inhaled corticosteroids (ICS), and phosphodiesterase-4 inhibitors (PDE4-Is), which aim to relieve symptoms and reduce exacerbations [50]. However, these therapies do not directly target the chronic inflammatory mechanisms driving COPD progression. Recent advancements in COPD treatment have introduced biologic agents such as Dupilumab (anti-IL-4Rα monoclonal antibody), Ensifentrine (a dual PDE3/4 inhibitor), and anti-IL-33 therapies [51–53], which have shown promise in controlling airway inflammation, particularly in patients with type 2 inflammation. Compared to these targeted biologic therapies, QJHTD exerts a broader anti-inflammatory effect by modulating multiple cytokines, including TNF-α, IL-6, and hs-CRP. While biologics selectively inhibit specific inflammatory pathways, QJHTD, as a multi-component herbal formulation, may provide a more comprehensive regulatory effect on both airway and systemic inflammation. However, no direct comparative studies have been conducted between QJHTD and these emerging COPD therapies. Further randomized controlled trials (RCTs) comparing QJHTD with both conventional pharmacological treatments and novel biologics are needed to determine its potential role as a complementary or alternative therapy in COPD management, particularly in patients with persistent inflammation despite standard treatments.

While there is no meta-analysis on the use of QJHTD for treating patients with stable COPD. Xing et al. [54] conducted a meta-analysis in China, which comprehensively evaluated the clinical efficacy of QJHTD for treating acute exacerbations of COPD (AECOPD). Their results indicated that QJHTD significantly improved the clinical efficacy in AECOPD patients and demonstrated good safety, which aligns with our findings. However, their analysis focused solely on AECOPD, while our study extends the investigation to stable COPD, specifically evaluating its effects on pulmonary function and inflammatory mediators. Despite these insights, previous studies have notable limitations that need to be addressed: (1) Previous studies focused on the therapeutic effects of QJHTD on AECOPD but did not evaluate its effects on stable COPD. (2) Previous studies did not conduct subgroup analyses, failing to consider the impact of treatment efficacy on different disease stages. (3) Previous studies only used the Cochrane risk of bias tool to assess the quality of the literature, without using the modified Jadad scale, potentially leading to insufficient quality assessment. (4) Previous studies assessed publication bias using only funnel plots and did not perform Begg’s and Egger’s tests, which might affect the reliability of the results. (5) Previous studies primarily focused on the efficacy and safety of QJHTD in treating COPD without exploring its effects on pulmonary function and inflammatory mediators. Pulmonary function and inflammation are closely related to disease prognosis, and our study successfully addressed this gap in the literature.

### Strengths and limitations

This study is the first systematic review and meta-analysis to evaluate the effects of QJHTD on pulmonary function and inflammatory mediators in patients with stable COPD. Inflammation is a primary pathogenic factor in stable COPD. It is associated with chronic inflammation of the airways, lung parenchyma, and systemic inflammation [7]. Inflammation leads to airway damage, airflow limitation, and airway remodeling. These processes result in decreased pulmonary function and disease progression [44]. Effectively suppressing inflammatory responses is crucial for controlling frequent COPD exacerbations and disease progression [47]. Previous studies have shown that QJHTD has anti-inflammatory and antioxidant effects in COPD treatment. It can reduce the decline in pulmonary function, and lessen disease exacerbations.

However, this study is not without limitations: (1) The included studies did not report specific allocation concealment and blinding methods. This introduces a high risk of implementation and especially measurement bias, particularly for subjective outcomes, which may compromise the internal validity and reliability of the results. (2) The overall quality of the included studies is relatively low. Although sensitivity and subgroup analyses were conducted, the sources of heterogeneity remain unidentified. This may be partly attributed to variations in treatment protocols, such as unstandardized dosages and durations of QJHTD administration. (3) Most studies did not report the severity of airflow limitation or specific baseline treatments of the subjects, making subgroup analysis based on these factors unfeasible. While a meta-analysis of pre- and post-treatment differences was performed to minimize inter-study variability, it may still impact the results. (4) All included studies were conducted in China, which may limit the generalizability of the findings to other populations. The lack of evidence from other regions raises concerns about external validity and limits the applicability of QJHTD in patients with different genetic backgrounds, lifestyles, and healthcare systems. (5) Potential publication bias is a noteworthy limitation. Most of the included studies were published in Chinese journals, which may lead to language bias and the exclusion of unpublished or non-significant findings. Although Begg’s and Egger’s tests were used to assess publication bias, the small number of included studies investigating TNF-α, IL-6, and hs-CRP limits the statistical power of these tests. Moreover, possible regional or institutional biases cannot be completely ruled out, given the homogeneous study origin. (6) The number of studies investigating TNF-α, IL-6, and hs-CRP was limited, providing insufficient supporting evidence. In addition, none of the included studies reported long-term outcomes such as sustained improvement in pulmonary function, frequency of exacerbations, or cost-effectiveness, which are crucial for real-world clinical application.

### Implication

To strengthen the evidence for QJHTD in treating stable COPD, future research should focus on the following areas: (1) Adherence to clinical research standards: Future studies should rigorously adhere to established clinical research protocols. They must clearly describe randomization procedures, allocation concealment, and especially the blinding of outcome assessors to minimize implementation and measurement bias, thereby improving the internal validity of trial findings. (2) Large-scale, multicenter clinical trials: Given that all current evidence is based on studies conducted in China, future research should involve large-scale, multicenter, and multinational clinical trials. This will provide more robust and generalizable evidence, helping to validate the efficacy and safety of QJHTD across populations with diverse genetic backgrounds, environments, and healthcare infrastructures. (3) Comprehensive and standardized outcome reporting: Future studies should not only report primary outcomes but also provide standardized descriptions of QJHTD dosage, duration, and baseline clinical characteristics, such as airflow limitation severity and prior treatments. This will enable more meaningful subgroup analyses and better assessment of clinical effectiveness. (4) Expanded evaluation of long-term and real-world outcomes: Beyond short-term clinical improvements, future trials should include long-term follow-up data on pulmonary function, acute exacerbation frequency, hospitalization rates, and cost-effectiveness to assess the sustained impact and real-world applicability of QJHTD. (5) Focused exploration of inflammatory mechanisms: Given the limited number and high heterogeneity of studies on inflammatory biomarkers, future research should conduct well-designed mechanistic studies with standardized methods to clarify the anti-inflammatory pathways through which QJHTD exerts its effects. (6) Comparative studies with standard and emerging COPD treatments: Given the recent advancements in COPD management, future research should explore how QJHTD compares with standard pharmacological treatments and novel biologics. Conducting head-to-head clinical trials assessing QJHTD’s efficacy alongside bronchodilators, ICS, and targeted biologics will help clarify its potential role in integrative COPD management.

## Conclusion

In conclusion, the current evidence suggests that QJHTD may improve pulmonary function, reduce inflammatory mediators, and enhance clinical efficacy as an adjunct therapy for stable COPD. It also demonstrates a favorable safety profile. However, these findings should be interpreted with caution due to several limitations, including small sample sizes, high heterogeneity, and methodological weaknesses such as unclear allocation concealment and lack of blinding. Future large-scale, multicenter RCTs with standardized protocols are needed to confirm these findings and further evaluate QJHTD’s role alongside stable COPD treatments.

## Supporting information

S1 Table Composition of Qingjin Huatan decoction. (DOCX)

S2 Table PRISMA 2020 checklist. (DOCX)

S3 Table Quality assessment of included studies. (DOCX)

S1 Appendix The search strategy. (DOCX)

S1 File All data extracted. (XLSX)

S2 File The excluded and included studies were listed. (XLSX)
