# Supplementary material for: Effect of Qingjin Huatan decoction on pulmonary function and inflammatory mediators in stable chronic obstructive pulmonary disease: A systematic review and meta-analysis
Source: PLoS One. 2025 May 7;20(5):e0322779. doi: 10.1371/journal.pone.0322779 (PMC12057979; doi:10.1371/journal.pone.0322779)
Supplement: S1 Table — (DOCX) [file pone.0322779.s001.docx]

# S1 Table. Composition of Qingjin Huatan decoction.

| No. | Chinese herbs | Latin name | Part of herbs |
| --- | --- | --- | --- |
| 1 | Huangqin | *Scutellaria baicalensis* Georgi | root |
| 2 | Zhizi | *Gardenia jasminoides* Ellis | fructus |
| 3 | Jiegeng | *Platycodon grandiflorum* (Jacq.) A. DC. | root |
| 4 | Maidong | *Ophio pogon japonicus* (L.f) Ker-Gawl | root |
| 5 | Sangbaipi | *Morus alba* L. | root bark |
| 6 | Zhebeimu | *Fritillaria thunbergii* Miq. | bulb |
| 7 | Zhimu | *Anemarrhena asphodeloides* Bge. | rhizome |
| 8 | Gualoupi | *Trichosanthes kirilowii* Maxim. | peel |
| 9 | Juhong | *Citrus reticulata* Blanco | peel |
| 10 | Fuling | *Poria cocos* (Schw.) Wolf | sclerotium |
| 11 | Gancao | *Glycyrrhiza uralensis* Fisch. | root and rhizome |
