# Supplementary material for: Effect of Qingjin Huatan decoction on pulmonary function and inflammatory mediators in stable chronic obstructive pulmonary disease: A systematic review and meta-analysis
Source: PLoS One. 2025 May 7;20(5):e0322779. doi: 10.1371/journal.pone.0322779 (PMC12057979; doi:10.1371/journal.pone.0322779)
Supplement: S3 Table — (DOCX) [file pone.0322779.s003.docx]

# S3 Table. Quality assessment of included studies.

| Study ID | Random sequence generation | Allocation concealment | Blinding | | Incomplete outcome data | Selective reporting | Other biases | Modified Jadad scores |
| --- | --- | --- | --- | --- | --- | --- | --- | --- |
|  |  |  | Blinding of participants and personnel | Blinding of outcome assessment |  |  |  |  |
| Cai et al. (2015) | Unclear risk | Unclear risk | Unclear risk | Unclear risk | Low risk | Low risk | Unclear risk | 3 |
| Hua. (2020) | High risk | Unclear risk | Unclear risk | Unclear risk | Low risk | Low risk | Unclear risk | 2 |
| Huang et al. (2020) | Unclear risk | Unclear risk | Unclear risk | Unclear risk | Low risk | Low risk | Unclear risk | 3 |
| Li and Wang. (2021) | High risk | Unclear risk | Unclear risk | Unclear risk | Low risk | Low risk | Unclear risk | 2 |
| Lin and Chen. (2016) | Low risk | Unclear risk | Unclear risk | Unclear risk | Low risk | Low risk | Unclear risk | 4 |
| Qi. (2021) | Low risk | Unclear risk | Unclear risk | Unclear risk | Low risk | Low risk | Unclear risk | 4 |
| Sun and Xu. (2020) | Low risk | Unclear risk | Unclear risk | Unclear risk | Low risk | Low risk | Unclear risk | 3 |
| Wan. (2019) | Unclear risk | Unclear risk | Unclear risk | Unclear risk | Low risk | Low risk | Unclear risk | 3 |
| Wang. (2023) | Low risk | Unclear risk | Unclear risk | Unclear risk | Low risk | Low risk | Unclear risk | 4 |
| Wu et al. (2023) | Low risk | Unclear risk | Unclear risk | Unclear risk | Low risk | Low risk | Unclear risk | 3 |
| Xia. (2021) | Low risk | Unclear risk | Unclear risk | Unclear risk | Low risk | Low risk | Unclear risk | 4 |
| Yi et al. (2018) | Low risk | Unclear risk | Unclear risk | Unclear risk | Low risk | Low risk | Unclear risk | 4 |
| Yu et al. (2015) | Unclear risk | Unclear risk | Unclear risk | Unclear risk | Low risk | Low risk | Unclear risk | 3 |
| Zhang and Zhang. (2015) | Low risk | Unclear risk | Unclear risk | Unclear risk | Low risk | Low risk | Unclear risk | 4 |
| Zhang. (2011) | Unclear risk | Unclear risk | Unclear risk | Unclear risk | Low risk | Low risk | Unclear risk | 3 |
| Zou et al. (2020) | High risk | Unclear risk | Unclear risk | Unclear risk | Low risk | Low risk | Unclear risk | 2 |
