# Supplementary material for: Effect of Qingjin Huatan decoction on pulmonary function and inflammatory mediators in stable chronic obstructive pulmonary disease: A systematic review and meta-analysis
Source: PLoS One. 2025 May 7;20(5):e0322779. doi: 10.1371/journal.pone.0322779 (PMC12057979; doi:10.1371/journal.pone.0322779)
Supplement: S1 Appendix — (DOCX) [file pone.0322779.s004.docx]

**S1 Appendix. The search strategy.**

**Search run on June 30 2024**

**PubMed (*n*=6)**

#1 ((Qingjin Huatan Decoction [Title/Abstract]) OR (Qingjin Huatan Tang [Title/Abstract]) OR (Qingjin Huatan Granules [Title/Abstract]))

#2 ((chronic obstructive lung disease [Title/Abstract]) OR (chronic obstructive pulmonary disease [Title/Abstract]) OR (chronic obstructive airway disease [Title/Abstract]) OR (COPD [Title/Abstract]))

#3 #1 AND #2

**Embase (*n*=3)**

#1 'Qingjin Huatan Decoction'/exp

#2 'Qingjin Huatan Tang'/exp

#3 'Qingjin Huatan Granules'/exp

#4 #1 OR #2 OR #3

#5 'chronic obstructive lung disease'/exp

#6 'chronic obstructive pulmonary disease'/exp

#7 'chronic obstructive airway disease'/exp

#8 'COPD'/exp

#9 #5 OR #6 OR #7 OR #8

#10 #4 AND #9

**Cochrane library (*n*=4)**

#1 MeSH descriptor: [Qingjin Huatan Decoction] explode all trees

#2 Qingjin Huatan Tang* or Qingjin Huatan Granules*.ti,ab,kw

#3 #1 or #2

#4 MeSH descriptor: [chronic obstructive pulmonary disease] explode all trees

#5 chronic obstructive lung disease* or chronic obstructive airway disease* or COPD*.ti,ab,kw

#6 #4 or #5

#7 #3 and #6

**Web of Science (*n*=3)**

#1 (TS=(Qingjin Huatan Decoction) OR ALL=(Qingjin Huatan Tang) OR ALL=(Qingjin Huatan Granules))

#2 (TS=(chronic obstructive pulmonary disease) OR ALL=(chronic obstructive lung disease) OR ALL=(chronic obstructive airway disease) OR ALL=(COPD))

#3 #1 AND #2

**CNKI (*n*=128)**

#1 篇关摘：清金化痰汤 OR 清金化痰颗粒

#2 篇关摘：慢性阻塞性肺疾病OR 慢阻肺OR 慢性气道阻塞性疾病 OR COPD OR 慢性气道阻塞

#3 #1 AND #2

**Wanfang Data (*n*=133)**

#1 主题：清金化痰汤 OR 清金化痰颗粒

#2 主题：慢性阻塞性肺疾病 OR OR 慢阻肺OR 慢性气道阻塞性疾病 OR COPD OR 慢性气道阻塞

#3 #1 AND #2

**CQVIP (*n*=90)**

#1 篇关摘：清金化痰汤 OR 清金化痰颗粒

#2 篇关摘：慢性阻塞性肺疾病 OR OR 慢阻肺OR 慢性气道阻塞性疾病 OR COPD OR 慢性气道阻塞

#3 #1 AND #2

**CBM (*n*=109)**

#1 常用字段：清金化痰汤 OR 清金化痰颗粒

#2 常用字段：慢性阻塞性肺疾病 OR OR 慢阻肺OR 慢性气道阻塞性疾病 OR COPD OR 慢性气道阻塞

#3 #1 AND #2

**中国临床试验注册中心 (*n*=2)**
